# Comparison of the accuracy of intraoral scans between complete-arch scan and quadrant scan

**DOI:** 10.1186/s40510-020-00337-1

**Published:** 2020-10-01

**Authors:** Youn-Gyeong Moon, Kyung-Min Lee

**Affiliations:** Department of Orthodontics, School of Dentistry, Chonnam National University, 33 Yongbong-ro, Buk-gu, Gwangju, 61186 South Korea

**Keywords:** Intraoral scan, Complete-arch scan, Quadrant scan

## Abstract

**Objective:**

To compare the accuracy of complete-arch scans and quadrant scans obtained using a direct chairside intraoral scanner.

**Material and methods:**

Intraoral scans were obtained from 20 adults without missing teeth except for the third molar. Maxillary and mandibular complete-arch scans were carried out, and 4 quadrant scans for each arch were performed to obtain right posterior, right anterior, left anterior, and left posterior quadrant scans. Complete-arch scans and quadrant scans were compared with corresponding model scans using best-fit surface-based registration. Shell/shell deviations were computed for complete-arch scans and quadrant scans and compared between the complete-arch scans and each quadrant scans. In addition, shell/shell deviations were calculated also for each individual tooth in complete-arch scans to evaluate factors which influence the accuracy of intraoral scans.

**Results:**

Complete-arch scans showed relatively greater errors (0.09 ~ 0.10 mm) when compared to quadrant scans (0.05 ~ 0.06 mm). The errors were greater in the maxillary scans than in the mandibular scans. The evaluation of errors for each tooth showed that the errors were greater in posterior teeth than in anterior teeth. Comparing the right and left errors, the right side posterior teeth showed a more substantial variance than the left side in the mandibular scans.

**Conclusion:**

The scanning accuracy has a difference between complete-arch scanning and quadrant scanning, particularly in the posterior teeth. Careful consideration is needed to avoid scanning inaccuracy for maxillary or mandibular complete-arch, particularly in the posterior area because a complete-arch scan might have potential error than a quadrant scan.

## Introduction

The introduction of an intraoral scanner improved on the many shortcomings of the traditional alginate impression using the tray and materials [1]. Intraoral scanning is now available for many branches of dentistry and performing a complete-arch scan directly in the patient’s mouth is more common in orthodontics.

The accuracy of intraoral scanners has been evaluated for both single tooth [2–8] and short-span-fixed dental prostheses [9–12]. To determine the accuracy of intraoral scanners, researchers have performed in vitro studies using reference models [13–18]. Although short-span intraoral scans have exhibited excellent accuracy, only a few studies have investigated the accuracy of in vivo intraoral scans for a complete-arch scan [19]. Considering that a complete-arch scan is more useful for orthodontic application than quadrant scan, research regarding the comparative accuracy between complete-arch scans and quadrant scans is needed. The purpose of the present study was to assess the accuracy of complete-arch scans and quadrant scans by standardizing the measurements using a reference model and then comparing these variances between the two different scans.

## Material and methods

The present study was approved by the Institutional Review Board of the Chonnam National University Dental Hospital, Gwangju, Korea. The inclusion criteria were (1) a fully erupted permanent dentition except for the third molars in both jaws, and (2) no prosthetic restorations such as metal crown or bridges on the molars.

**Fig. 1:**
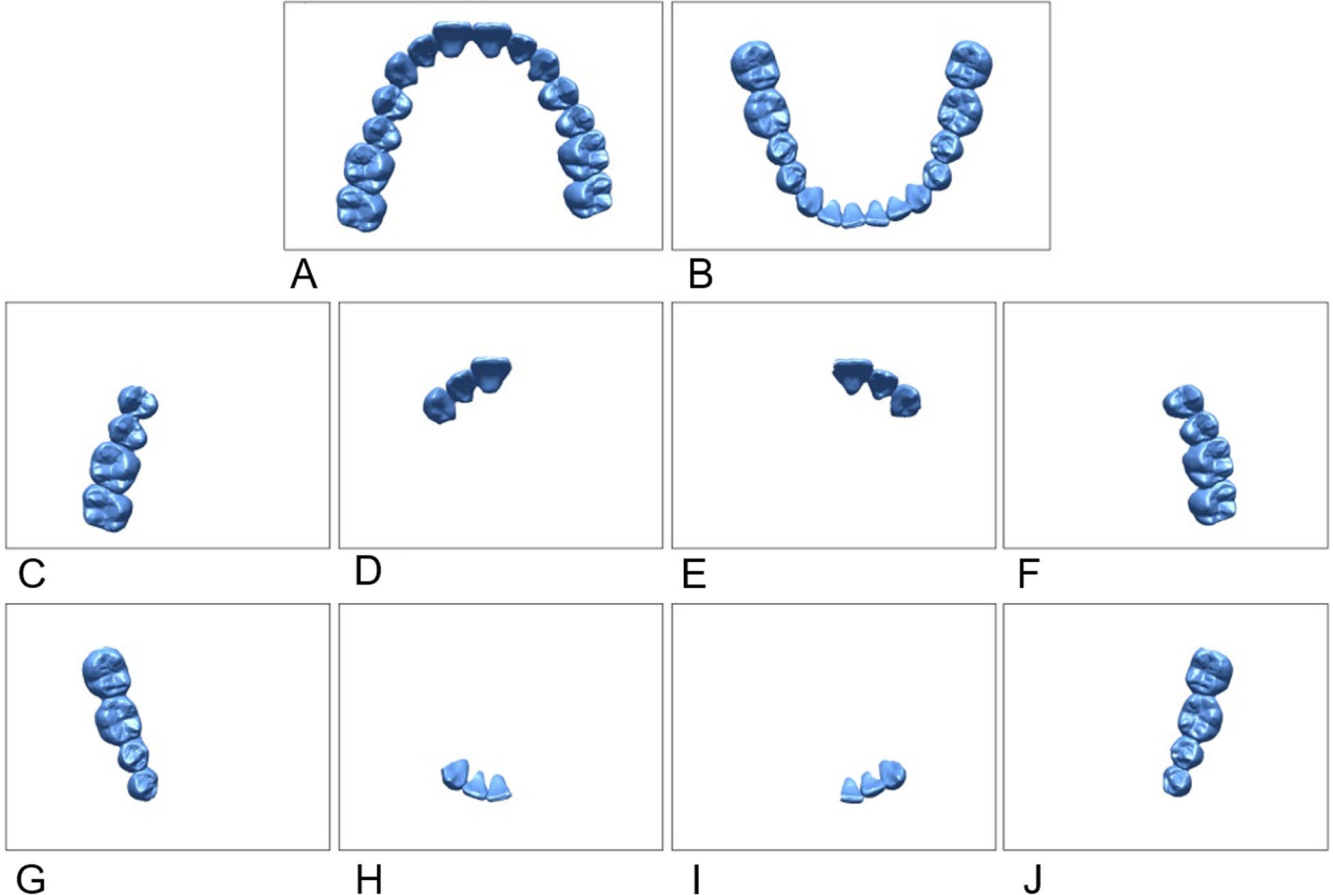
Intraoral scans obtained from each patient. **a**, **b** Complete-arch scan; **c**–**j** Quadrant scan

Twenty patients had complete-arch scans with an intraoral scanner (TRIOS, 3Shape, Copenhagen, Denmark). In addition, 4 quadrant scans were obtained from the right posterior, right anterior, left anterior, and left posterior areas for each arch. The scan data were reprocessed as a stereolithography file format by using OrthoAnalyzer^TM^ software program (3Shape) (Fig. 1).

A model scan was used as the gold standard to compare accuracy of the complete-arch scans and quadrant scans. Maxillary and mandibular alginate impressions (Cavex Impressional, Cavex Holland BV, Haarlem, the Netherlands) were taken and immediately poured with dental stone (New Plastone II White, GC Corporation Tokyo, Japan). The models were then scanned by using a desktop scanner (Orapix, Seoul, Korea), and scan files were converted to 3D (three-dimensional) images by a reverse engineering software program (Rapidform, 3D Systems, Rock Hill, SC).

**Fig. 2:**
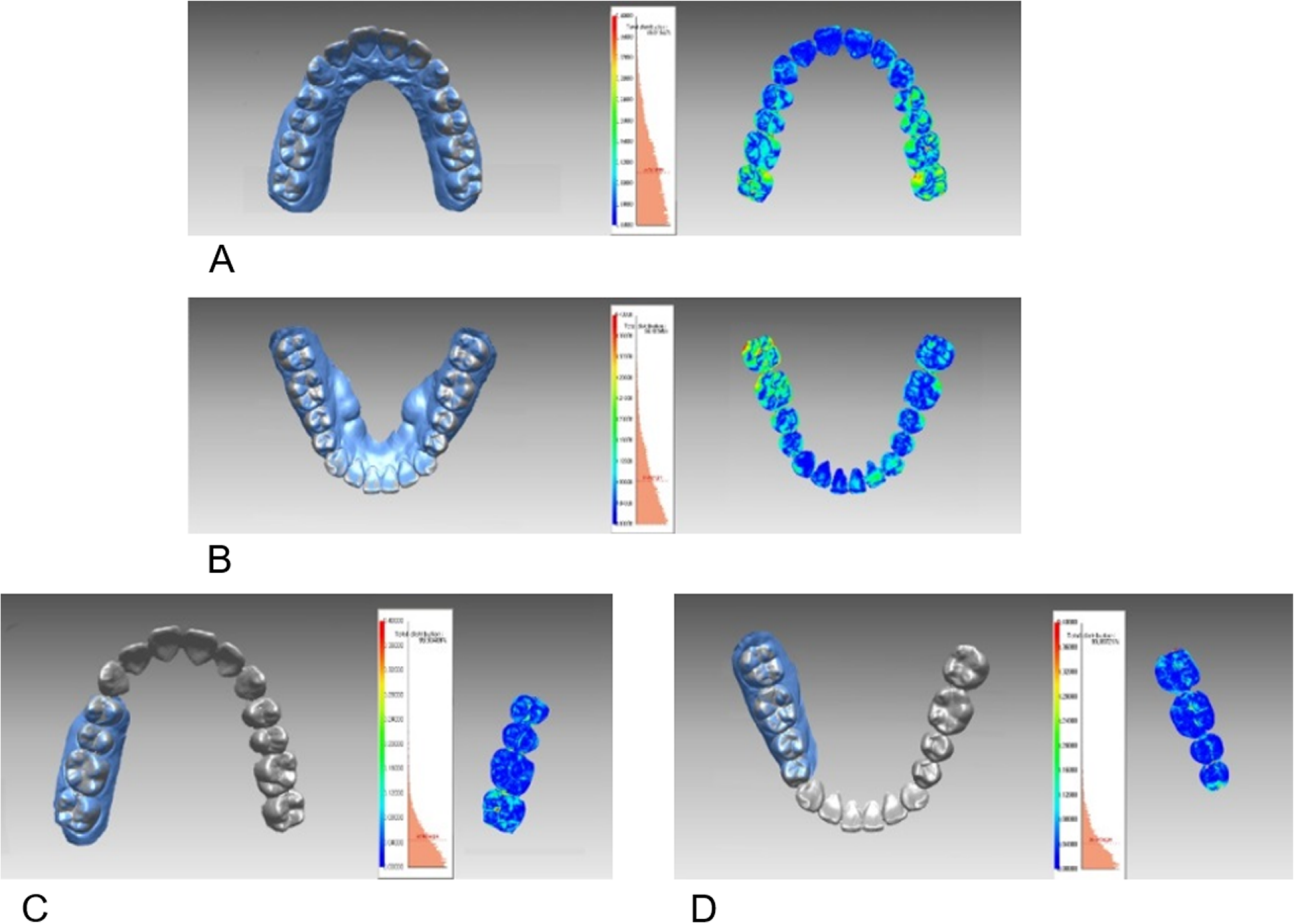
Three-dimensional superimposition with the laser-scanned image and color-coded visualization charts showing shell/shell deviation in complete-arch scan (**a**, **b**) and quadrant scan (**c**, **d**) images. Right posterior scan images were presented as an example of quadrant scan images

Each complete-arch scans and quadrant scans were superimposed with a model scan using best-fit surface feature-based registration. The initial registration involved the selection of three corresponding points in each of the two scans, after which the program’s automatic fine registration function was employed to finalize the matches. In a complete-arch scan, incisal midpoint and mesiobuccal cups of the right and left first molar were used as three reference points. The buccal cusp of the first premolar and lingual cusp of the second premolar, and the mesiobuccal cusp of the second molar were used in the quadrant posterior scan. The canine cusp, lingual zenith of lateral incisor, and labial zenith of central incisor were used in the quadrant anterior scan. Using the “shell/shell deviation” function in the program, the average surface differences between intraoral scan and the model scan were computed at all points on the surfaces. The average surface differences were calculated also for each individual tooth in complete-arch scans to evaluate factors which influence the accuracy of intraoral scans. In addition, these differences were visualized by means of color-mapping chart (Fig. 2).

### Statistical analysis

The calculation of sample size was based on the previous study [20]. Shell/shell deviations were computed for complete-arch scans and quadrant scans and compared between the complete-arch scan and each quadrant scan using the paired *t* test. ANOVA was used to detect differences among 4 quadrant scans for each arch. Also, a paired *t* test was used to detect the significant difference between complete-arch scans and quadrant scans. The statistical analyses were performed using the SPSS software package (version 23.0; IBM, Armonk, NY).

## Results

**Fig. 3:**
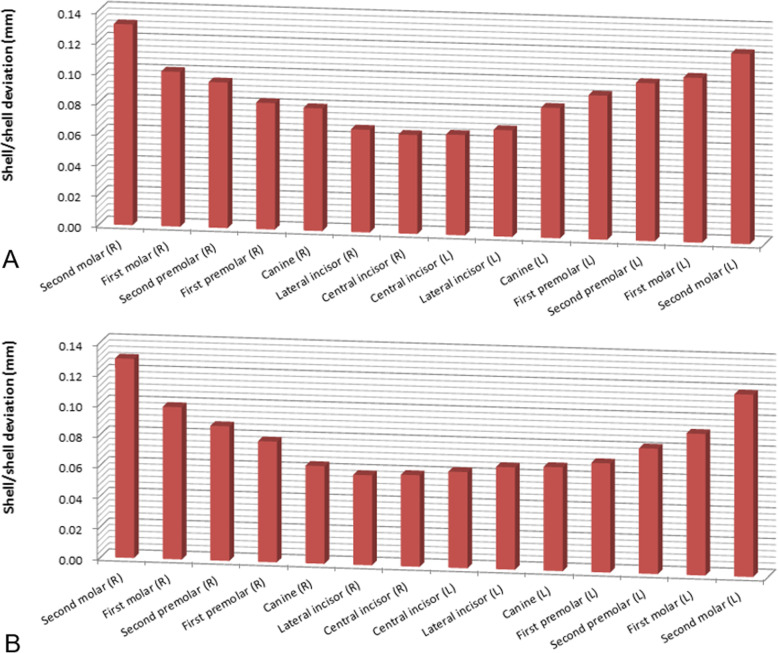
Graphic presentation of shell/shell deviation of individual tooth in complete-arch scan. **a** Maxilla. **b** Mandible

The errors of the complete-arch scans and model scans showed 0.10 mm in maxillary scans and 0.09 mm in mandibular scans. The errors were larger in the maxillary scan images than in the mandibular scan. In the case of quadrant scans, the errors ranged from 0.05 to 0.06 mm. For each segmental area, the differences in the errors were statistically significant in both arches (Table 1). The complete-arch scan showed more variation than the quadrant scan in both arches. The evaluation of the individual teeth showed that the variation was greater in the posterior teeth than in anterior teeth. The right side of the posterior teeth showed a greater variation than the left side in the mandibular arch scans; however, there was no significant difference between the right and left sides (Fig. 3).

**Table 1:** Average surface difference with reference model scan in each complete-arch scan and quadrant scan (mm)

	*Complete-arch scan*	*Quadrant scan*				
		*Right posterior*	*Right anterior*	*Left anterior*	*Left posterior*	*Difference*
	*Mean ± SD*	*Mean ± SD*	*Mean ± SD*	*Mean ± SD*	*Mean ± SD*	
Maxilla	0.10 ± 0.02	0.06 ± 0.01	0.05 ± 0.02	0.05 ± 0.01	0.06 ± 0.02	*P* < 0.05
Mandible	0.09 ± 0.01	0.06 ± 0.02	0.05 ± 0.01	0.05 ± 0.01	0.06 ± 0.02	*P* < 0.05
Difference	*P* < 0.05	*P* = 0.895	*P* = 0.527	*P* = 0.847	*P* = 0.645	*P* < 0.05

*SD* standard deviation

## Discussion

Different branches of dentistry require different levels of variance when scanning is compared to the alginate impression. For instance, a 0.12 mm margin discrepancy has been reported to be the limit for a clinically acceptable crown margin in prosthetics [21], whereas for orthodontics, a difference in tooth size measurement within 0.27 mm was reported to be clinically insignificant [22]. Although silicone-based impression materials are known to be more precise than alginate impression, the alginate impression is generally used for the diagnosis of patients in orthodontic clinics. In this study, the alginate impression was used for comparing a complete-arch scan and quadrant scan.

The results of this study showed that the errors of the full arch scans were statistically more significant than those of the quadrant scans. The largest difference from the quadrant scans was 0.04 mm. This finding is clinically acceptable, considering that the result also reflects the variance in the impression acquisition and the manufacturing process of the plaster model. The errors in the posterior areas were larger than those in the anterior area. One possible explanation for this finding is that there is more saliva in the posterior areas of the mouth. The dryness of the tooth during scanning is critical for scanning accuracy. Additionally, the scanning sequence and the process of stitching the scanned images may affect the accuracy in the molar areas. TRIOS systems captured single images of the tooth and produced an assembled virtual model of the whole dentition. Smaller tooth surfaces, those seen in incisors, have limited overlap area between captured images. Scanning and stitching can accumulate variance in the scans. This variance was more pronounced in the mandibular arch than in the maxillary arch, which was thought to be inaccuracy due to the tongue during mandibular arch scanning. In other words, the variance introduced during stitching may be more likely to occur in the mandibular arch than in the maxillary arch. When we looked at the variation between left and right in this study, the error tended to be larger on the right side than on the left side, although the difference was not statistically significant. This difference is also thought to be a result of the scanning direction. Scanning direction may also be a factor in these differences. In this study, scanning started from the left side of the arch, based on the manufacturer’s recommendation. As the scanner moved from the left side to the right, the variances might have accumulated on the right side and in the posterior areas.

The results suggest entirely removing saliva during the scans to increase the accuracy of complete-arch scans when compared with the quadrant scans. If careful adjustment is made to reduce the stitching error, such as scanning the labial or lingual side of the incisal edge possible during anterior scanning, this will help to improve the accuracy of the complete-arch scans. Accuracy can also be affected by the clinician’s skill during intraoral scanning. In the present study, the intraoral scans were obtained by the same examiner experienced in intraoral scanning with over 100 patients. Scanning times tend to decrease as operator experience increased. Long scanning time may exacerbate variance due to the stitching process of captured images.

The scanning accuracy has a difference between complete-arch scanning and quadrant scanning, particularly in the posterior teeth. The error was greater in the posterior teeth than in the anterior teeth. The errors in the right side posterior teeth appeared a substantial variance than the errors on the left side when the scanning was started from the left side. Based on the results, clinicians should pay more attention to the accuracy of the complete-arch scanning when performing intraoral scanning for the purpose of orthodontic treatment, particularly for the fabrication of removable appliances. Careful consideration is needed to avoid scanning inaccuracy for maxillary or mandibular complete-arch, particularly in the posterior area because a complete-arch scan might have potential error than a quadrant scan.

## Conclusion

The scanning accuracy has a difference between complete-arch scanning and quadrant scanning, particularly in the posterior teeth. Careful consideration is needed to avoid scanning inaccuracy for maxillary or mandibular complete-arch, particularly in the posterior area because a complete-arch scan might have potential error than a quadrant scan.

## Data Availability

The STL files and the 3D surface models obtained in this study belong to the authors and are therefore available only upon request, after approval by the authors.

## Abbreviations

3D: Three-dimensional
